# Correction: Xixi, Li., et al. The Plastidial Glyceraldehyde-3-Phosphate Dehydrogenase Is Critical for Abiotic Stress Response in Wheat. *Int. J. Mol. Sci.* 2019, *20*, 1104

**DOI:** 10.3390/ijms21124499

**Published:** 2020-06-24

**Authors:** Xixi Li, Wenjie Wei, Fangfang Li, Lin Zhang, Xia Deng, Ying Liu, Shushen Yang

**Affiliations:** College of Life Sciences, Northwest A&F University, Yangling 712100, Shaanxi, China; lixixi2018wlf@163.com (X.L.); wenjiewei2011@163.com (W.W.); liffnwafu@163.com (F.L.); Lin798335901@163.com (L.Z.); 18209272902@163.com (X.D.)

The author wishes to make the following correction to this paper [1]. Due to mislabeling of Figure 6a, replace:

**Figure 6a:**
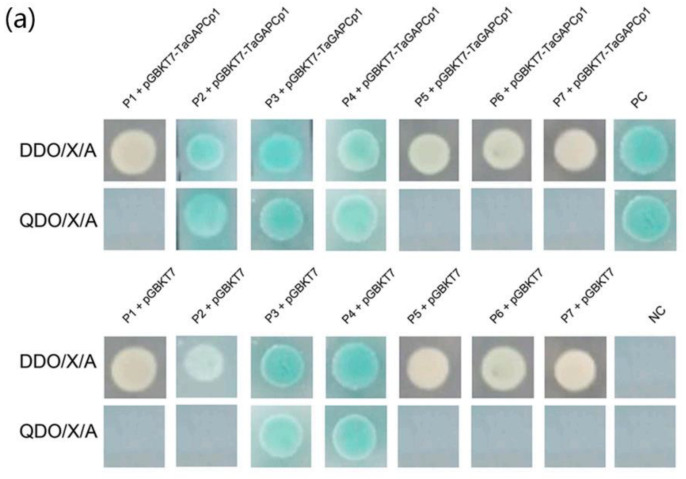


with

**Figure 6a:**
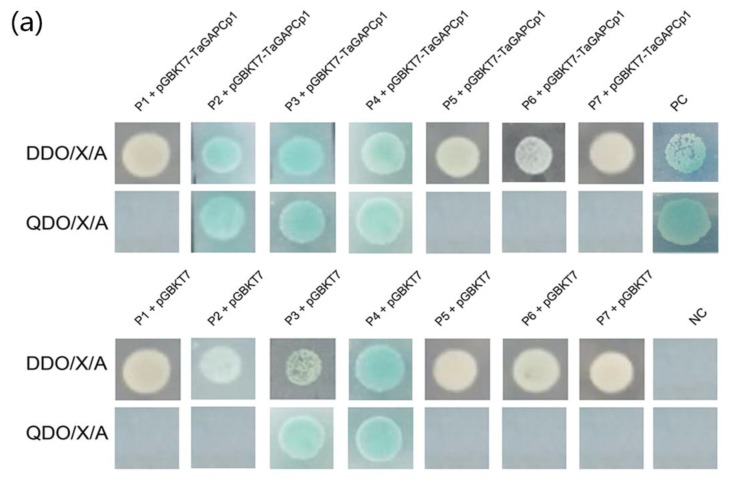


The authors would like to apologize for any inconvenience caused to the readers by this change.
