# Supplementary material for: The Plastidial Glyceraldehyde-3-Phosphate Dehydrogenase Is Critical for Abiotic Stress Response in Wheat
Source: Int J Mol Sci. 2019 Mar 4;20(5):1104. doi: 10.3390/ijms20051104 (PMC6429432; doi:10.3390/ijms20051104)
Supplement: Supplementary file 1 [file ijms-20-01104-s001.pdf]

International Journal of
Molecular Sciences

MDPI

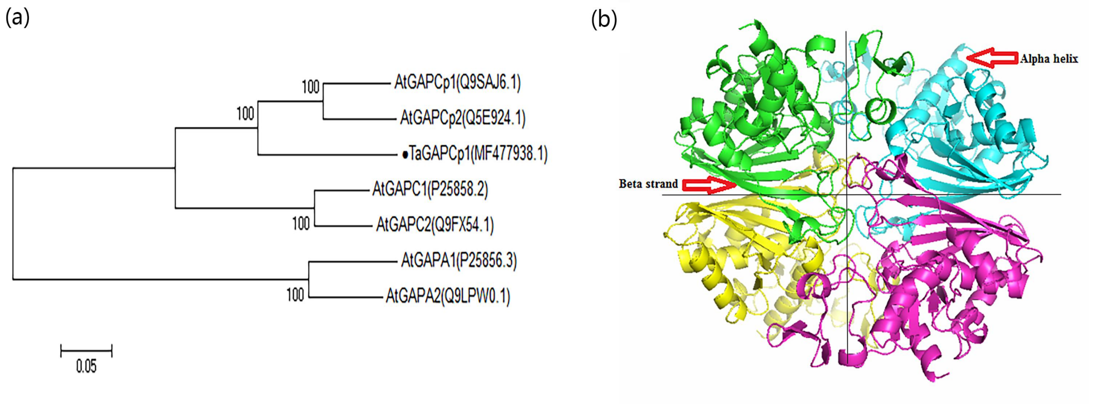

Fig. S1 Phylogenetic analysis and structural analysis of TaGAPCp1. (a) The phylogenetic tree was constructed with MEGA6.0 using the neighbor-joining(NJ) method. Numbers above or below branches of the tree indicate bootstrap values. The values in parentheses represent the accession number of GAPDH. **(b)** The spiral portion is Alpha helix and the arrow portion is Beta strand. Alpha helix account for 25% and Beta strand account for 30%

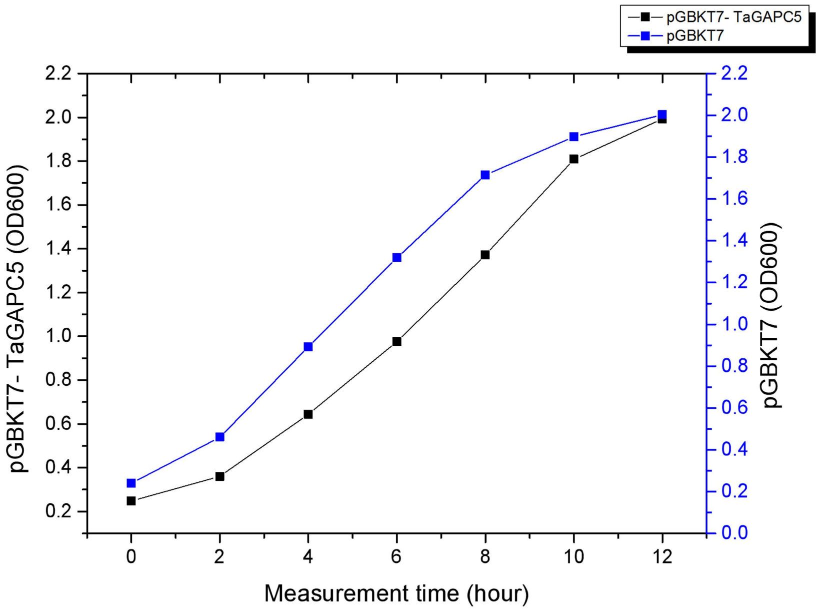

Fig. S2 Toxicity detection of pGBKT7-GAPCp1 against yeast. Whether pGBKT7-GAPCp1 is toxic to yeast by concentration determination after liquid culture of yeast containing pGBKT7-GAPCp1 and pGBKT7, respectively

Table S1. Names and sequences of primer

Primer name	Sequences (5′→3′)
TaGAPCp1-F	AGGCCGAATTCCCGGGGATCCGTATGGCCTATATGTTCAAGTATG
TaGAPCp1-R	CCGCTGCAGGTCGACGGATCCTCGGAGGTGATCAGTGCTT
qRT-PCR-F-TaGAPCp1	CACCCGCTCCGCTCATTTTG
qRT-PCR-R-TaGAPCp1	AAGTGGCTCAATGTTCCTGG
β-actin-F	CGACTCTGGTGATGGTGTGAG
β-actin-R	AGCAAGGTCCAAACGAAGGA

1302-F	ACGGGGGACTCTTGACCATGGATGGCCTATATGTTCAAGTATG
1302-R	TACTAGTCAGATCTACCATGGGTGCTTGGCGGAGACAAGAGC
N-TaGAPCp1-F	CCCAGGCCTACTAGTGGATCCATGGCCTATATGTTCAAGTATG
N-TaGAPCp1-R	ACCCTCGAGGTCGACGGATCCTCGGAGGTGATCAGTGCTT
C-Cyt b6f-F	TGGCGCGCCACTAGTGGATCCATGGCCTCTACCGCGCT
C-Cyt b6f-R	GACAGTACTATCGATGGATCCTTTCCACCACGGGTTGTC
qRT-PCR-F-Cyt b6f	CACCGCCTCCAACCCTA
qRT-PCR-R-Cyt b6f	GGCCATGCAGGTGATGTT

Table S2. Gene CDS sequence

TaGAPCp1 cds（Traes_6AS_D1274A812.1）：

ATGGCCTATATGTTCAAGTATGACTCCACTCATGGCCCCTTTAAAGGATCCATCAATGT
TGTGGATGATTCAACCCTGGAGATCAATGGGAAGAAAATCACTATAACAAGCAAAAG
AGATCCCGCAGAGATTCCTTGGGGTAACTTTGGAGCTGACTATGTTGTTGAATCTTCTG
GTGTTTTTACAACCATCGATAAAGCATCGGTACACTTGAAGGGTGGTGCTAAGAAAGT
GGTGATATCTGCTCCATCCGCAGATGCTCCCATGTTTGTGGTTGGAGTAAATGAGATGA
GCTACGATCCCAAGATGAATGTTGTTTCTAATGCAAGTTGTACCACTAACTGTCTTGCT
CCTCTTGCCAAGGTTGTTCATGAAGAATTTGGCATTCTTGAAGGTCTAATGACAACTGT
TCACGCCACAACAGCCACCCAAAAGACTGTTGATGGTCCTTCAATGAAAGATTGGAGA
GGAGGGCGTGGTGCTGGTCAAAACATAATTCCTAGCTCCACTGGTGCAGCAAAGGCTG
TTGGGAAAGTTCTCCCTGAGCTAAATGGAAAGCTTACTGGCATGGCTTTCCGAGTTCCA
ACACCAAATGTCTCTGTGGTTGACTTGACCTGCCGGCTTGAGAAAAGTGCCTCTTATGA
TGATGTGAAAGCAGCCATCAAGGCGGCATCAGAGGGTGCACTGAAAGGCATTTTAGG
CTACACAGATGAGGATGTGGTATCCAACGATTTTGTTGGTGACACAAGGTCAAGTGTA
TTTGATGCTAATGCTGGCATGGGGCTGAGCTCTTCCTTCATGAAGCTCGTGTCGTGGTA
CGACAACGAGTGGGGCTACAGCAACCGGGTTCTTGATCTCATCGCCCACATGGCTCTT
GTCTCCGCCAAGCACTGA

Cyt b6f cds (XM_020345741.1)

ATGGCCTCCACCGCGCTCTCCACCGCCTCCAACCCTACCCAGCTCTGCCGGACCCGCG
CAAGCTCGCTGTGCAAGCCCGTCAAGGGCCTGGGCTTCGGCCGGGAGCGCGTCCCGAG
GAACATCACCTGCATGGCCGGCAGCATCTCCGCGGACCGCGTGCCGGACATGAGCAA
GAGGGAGCTGATGAACCTGCTCCTGCTGGGCGCCATCTCGCTCCCCACCTTCGGCATG
CTCGTCCCCTACGGCTCCTTCCTCGTCCCGGCCGGCTCCGGGAGCAACGCCGGAGGGG
TCGCCGCCAAGGACAAGCTCGGCAACGACATCCTCGTCGAGGACTGGCTCAAGACGC
ACGGCCCCAACGACCGCACGCTCGCCCAGGGGCTCAAGGGTGATCCTACCTACCTTGT
GGTGGAGTCCGACAAGACCCTCGCCACCTACGGGATCAACGCCGTGTGCACGCATCTT
GGATGCGTCGTGCCGTGGAACGCCGCCGAGAACAAGTTCCTCTGCCCCTGCCATGGAT
CCCAGTACAACAACCAGGGCAAGGTTGTCCGTGGGCCTGCACCTCTGTCGCTGGCCCT
TGTTCACGCCGACGTCGACGACGGCAAGGTCGTCTTCGTGCCGTGGGTGGAGACCGAC
TTCAGGACCGGCGACAACCCGTGGTGGAAATAA

Tauschii Dormancy-Associated Protein Homolog cds (GenBank XM_020318726.1)
ATGGGGCTCCTCGACCAGCTCTGGGACGAGACGGTCGCCGGCCCCAGGCCGGACCAC
GGCCTCGGCAGGCTCCGCAAGTACTCCTCCTTCTCGCCCTCGACCGCGGCGGCCACGG
ACGTCGCGCCGGCGGTGACCCGCAGCATCACCATCGCCCGGCCGCCATCTCTCTCGGT
GCCATCTGGCGAATCAAGCTCCTTGCCGCCGTCCCCGGCCAGCGCGCCGGACTCCCCG
TTCGCAGCAGCTACCAGTAGGAGGGACGGCTGGAGGGCGTTCAGGCGGAAATCCAAG
ATGGCCAACGTCGACGTCGTCCGGCCTGAGACGGCCGTCGGGCCCAGAAGCCCCACC
GTGTACGACTGGGTGGTGATCAGTTCATTGGACCGATGA

Tauschii ATP-Dependent Clp Protease Proteolytic subunit cds (GenBank XM_020331538.1)
ATGGCCATCCCCACGGCCCTCGCCGTCCGCGCCTCTCCCTCCTGCCAATTCTCGCACAG
TTCGTCCTTTGCCTGCTCTCCTTTGTCTAGGCGGAATGGAAGCAGTGCATCTCGGCGCCT
CCGAGGACTGCAAGTTTTAACATCAGGTGTTTCTGCACGGTTGGATGAGAGGATACAC
AGTCAATCTTATCCGAGGAAAAATACAATCGTGGCATCAGAGAATGAAAATCCACCTT
TAATGCCTGCTATCATGACTCCTGGTGGCCCTCTGGATCTGGCAACCGTACTGCTAGGA
AACCGTATAATCTTCATTGGTCAATATGTTAACTCGCAAGTGGCACAACGTGTGATATC
ACAGCTTGTCACACTTTCTGCTATAGATGAAGAGGGTGATATTCTGATCTATCTTAACT
GCCCTGGCGGAAGTCTCTATTCTATCTTAGCAATTTATGATTGCATGTCCTGGATCTCAC
CAAAAGTTGGGACAGTGTGCTTTGGTGTTGTTGCTAGTCAAGCAGCAATCATTCTTGCT
GGTGGTGAGAAGGGGATGCGCTATGCGATGCCTAATGCCAGAGTAATGATTCATCAGC
CTCAAGGTGGATCGGAGGGTAATACGGAGGAGGTGAGGAGACAGGTTGGGGAAACC
ATTTATGCTCGAGATAAAGTTGACAAAATGTTTGCTTCTTTCACGGGGCGACCCATCGA
TATAGTGCGACAGTGGACAGAGAGGGATCGTTTCATGTCGTCGTCCGAGGCCATGGAC
TTCGGACTAGTCGACGCATTGCTGGAAACGGGATGCTAA

Tauschii Ribulose-1,5-Bisphosphate Carboxylase (*RUBP*) cds (GenBank KT288199.1)
ATGGCCCCCACCGTGATGGCCTCGTCGGCCACCTCCGTCGCTCCTTTCCAGGGGCTCAA
GTCCACCGCCGGCCTCCCCGTCAGCCGCCGCTCCAACGGCGCTAGCCTCGGCAGCGTC
AGCAACGGTGGAAGGATCAGGTGCATGCAGGTGTGGCCCATCGAGGGCATCAAGAAG
TTCGAGACCCTGTCTTACCTGCCACCGCTCAGCACGGAGGCCCTCCTCAAGCAGGTCG
ACTACCTGATCCGCTCCAAGTGGGTGCCTTGCCTCGAGTTCAGCAAGGTTGGGTTCATC
TTCCGTGAGCACAACGCATCTCCTGGGTACTACGATGGCCGGTATTGGACAATGTGGA
AGCTGCCTATGTTCGGGTGCACTGACGCCACACAGGTGATCAACGAGGTGGAGGAGGT
CAAGAAGGAGTACCCTGACGCGTATGTCCGCATCATCGGATTCGACAACATGCGTCAG
GTGCAGTGCGTCAGCTTCATCGCCTTCAAGCCACCGGGCTGCGAGGAGTCCGGCAAGG
CCTAA

Tauschii GATA Transcription Factor (*GATA*) cds (GenBank XM_020324318.1)
ATGGATTCGTCTCACCACAAGGTGATCGGGGTCGCTCCGGCGGCGGCGGAGGGAGGG
AGGATGTGCTGCGTCGAATGCCGCACCACCACCACTCCCATGTGGCGCAGCGGGCCAA
CGGGGCCGAGGACGCTTTGCAACGCGTGCGGGATCCGATACAGGAAGAAGAGGAGGC

AAGAGCTAGGTCTCGACAACAAGCCGCAGAACCAGCAGCTGAACCAACAACAACAA
CAGCAGCAGCAGCAGCAACAACAACAACAACAACAAGAAGATCACGGAGAGGCAA
CAAGTGCAGTAAAAGACAGCAGCAGCAGTACCAACAACAAAAGCAACAGTCTCCAA
GTGGTGAAGAAGAAAAGGACGGTCTCAATGGGGGTGGAGGAGGCTGCATTCTTGCTC
ATGGCCTTGTCCTCCTCATCTACACCCCCACTACTACATGGCTGA

Tauschii Probable E3 Ubiquitin-Protein Ligase cds (GenBank XM_020328679.1)
ATGTCAGGAGGTCGGCAGACATATTGGTGCTTCCAGTGTAGACAGCGGGTTAGGCCGC
GTGGACGGGAGATGGAGTGTCCTTACTGTGATGCTGGCTTTGTGGCTGAAATGGATGAT
GTCGATGCTCTCATGAGCCAATTTGTTGGGATGTATAGTGATTTTCATCGTGACCCAAG
GTTCGGGATCATGGAGGCAATGTCTACCGTGATGCGACATGGAATGGGGAGCATGAAT
CGAGAGGCTGATGTAAGAGGAAGGCCAAGCATATTATCTGACCTGGAGACGGAATTT
GGTTCAGGGCCATGGTTGCTCTTCCGTGGCCAGCTCCCCGGTCATCTCTCAGAGGCCAA
TAGTGGTTTCGACGTTTTCGTCAATGGACGCCGTGGTGTTGGCATGCGGAGGGCAGATG
TTGCAGATTACTTTGTTGGGCCTGGATTGGAAGATCTGATTGAGCAGTTGACTCATAAT
GATCGTCGAGGGCCACCACCTGCCTCTCAGTCATCGATTGATGCCATGCCTAGTGTTAG
GATCACTGCCAGGCATCTCACTGGAGACTCGCATTGCCCTGTCTGCAAGGACAAGTTT
GAACTGGGATCAGAAGCAAGAGAGATGCCATGCAAGCATTTATACCACTCTGATTGCA
TACTTCCTTGGCTAGAGCAACACAATTCCTGTCCTGTTTGCCGATATGAGCTGCCCACA
CAGAGCTCTAGTGGTGCCAGCTGCTCACGCTCAAGATCAACCAACCAAAATGACAGTT
CAAGCAGCTCAAGTAGCAGTGGGAGAACCAGCGGGCGTCAGAGGAGAAGGAACCCG
TTCTCGTTCCTATGGCCTTTCCGCTCATCAAGTTCTAGCACTGGTTCTCGTTAG

Tauschii Protein *SGT1* cds (GenBank KJ907387.1)
ATGGCCGCCGCCGCCGCGTCGGATCTGGAGAGCAAGGCCAAGGAGGCCTTCGTCGAC
GACGACTTCGAGCTGGCCGCCGAGCTCTACACCCAGGCCATTGAGGCGGGGCCGGCC
ACCGCGGAACTCTACGCCGACCGAGCCCAGGCGCACATCAAGCTGGGCAGTTACACT
GAGGCTGTAGCTGATGCCAACAAAGCAATTGAACTTGATCCTTCGATGCATAAAGCAT
ACCTTCGGAAGGGCTCTGCTTGCATCAAGCTGGAGGAATACCAAACTGCAAAGGCTGC
TCTTGAAGTGGGTTCTTCATATGCATCTGGTGACTCGAGGTTTACTCGTCTGATGAAGG
AGTGTGATGATCGTATTGCTGAGGAGGCTAGCCAGGTGCCAGTAAAGAATGCCGCTGC
AGCTGTTGCTCCAGCTACATCTTCGGGGGCATCTTCCGGGGCTACAACTGTGGCTACTG
AAGTTGAGGACCAGGATGGTGCAAATATGGAGAATGCACAGCCAACGGTAGAAGTGC
CAAGCAAGCCCAAATACAGGCATGACTACTACAATACTCCTACAGAAGTGGTACTGA
CTATATTTGCTAAGGGTGTTCCAGCTGACAGCGTGGTTGTTGACTTTGGTGAACAGATG
CTGAGTGTCTCAATTGAACTTCCCGGTGAGGAACCATACCATTTTCAGCCTCGTCTGTTT
TCAAAGATCGTCCCAGATAAGTGCAAGTATACTGTGTTGTCTACAAAGGTTGAAATGC
GCCTTGCAAAAGCTGAGCCAGTAACTTGGACATCATTGGATTATACTGGTAAACCAAA
GGCTCCTCAGAAGATAAATGTACCAGCTGAATCAGCCCAGAGGCCATCTTATCCATCA
TCAAAATCCAAAAAGGACTGGGATAAGCTTGAAGCTGAAGTGAAAAAACAGGAGAA
GGATGAAAAACTTGATGGCGATGCTGCATTGAACAAATTTTTCCGTGAAATTTACAGT
GATGCTGATGAAGATATGCGTAGAGCAATGATGAAGTCTTTTGTGGAGTCTAATGGAA

CCGTTCTCTCAACCAACTGGAAGGATGTCGGGAAAAAGACGGTTGAAGGAAGCCCTC
CTGATGGAATGGAGCTCAAGAAGTGGGAGTATTAA

© 2019 by the authors. Licensee MDPI, Basel, Switzerland. This article is an open access article distributed under the terms and conditions of the Creative Commons Attribution (CC BY) license (http://creativecommons.org/licenses/by/4.0/).
